# Preeclampsia Drives Molecular Networks to Shift Toward Greater Vulnerability to the Development of Autism Spectrum Disorder

**DOI:** 10.3389/fneur.2020.00590

**Published:** 2020-07-15

**Authors:** Qinglian Xie, Zhe Li, Yan Wang, Shan Zaidi, Ancha Baranova, Fuquan Zhang, Hongbao Cao

**Affiliations:** ^1^Department of Outpatient, West China Hospital of Sichuan University, Chengdu, China; ^2^Mental Health Center and National Clinical Research Center for Geriatrics, West China Hospital of Sichuan University, Chengdu, China; ^3^School of Systems Biology, George Mason University, Fairfax, VA, United States; ^4^Research Centre for Medical Genetics, Moscow, Russia; ^5^Department of Psychiatry, The Affiliated Brain Hospital of Nanjing Medical University, Nanjing, China; ^6^Department of Psychiatry, First Hospital/First Clinical Medical College of Shanxi Medical University, Taiyuan, China

**Keywords:** autism, autism spectrum disorder, preeclampsia, GSEA, extreme male brain theory

## Abstract

Preeclampsia (PE) confers a significant risk for subsequent diagnosis with autism spectrum disorder (ASD), with the mechanisms underlying this observation being largely unknown. To identify molecular networks affected by both PE and ASD, we conducted a large-scale literature data mining and a gene set enrichment analysis (GSEA), followed by an expression mega-analysis in 13 independently profiled ASD datasets. Sets of genes implicated in ASD and in PE significantly overlap (156 common genes; *p* = 3.14E^−67^), with many biological pathways shared (94 pathways; *p* < 1.00E^−21^). A set of PE-driven molecular triggers possibly contributing to worsening the risk of subsequent ASD was identified, possibly representing a regulatory shift toward greater vulnerability to the development of ASD. Mega-analysis of expression highlighted RPS4Y1, an inhibitor of STAT3 that is expressed in a sexually dimorphic manner, as a contributor to both PE and ASD, which should be evaluated as a possible contributor to male predominance in ASD. A set of PE-driven molecular triggers may shift the developing brain toward a greater risk of ASD. One of these triggers, chromosome Y encoded gene RPS4Y1, an inhibitor of STAT3 signaling, warrants evaluation as a possible contributor to male predominance in ASD.

## Introduction

Autism spectrum disorders (ASD) are a range of neurodevelopmental mental disorders affecting nearly 1% of the global population (62.2 million globally) (1) and more than 2% of children (about 1.5 million) in the United States of America (www.hrsa.gov). Studies in recent years revealed hundreds of genes linked to ASD, paving the way for understanding the pathological mechanisms of the disease (2–4).

Preeclampsia (PE) is a disorder of pregnancy that increases the risk of poor outcomes for both the newborn and the mother. In PE, patients commonly present with high blood pressure, then their condition aggravates with the reduction *in utero* placental blood flow (1). Recent studies indicate that PE is one of the important risk factors for ASD (5, 6). However, the underlying mechanisms are largely unknown.

ASD and PE do share at least some pathophysiological pathways. For example, one of the core clinical features of PE is a systemic inflammation in a mother (Shennan et al., 2015). In turn, prenatal exposure to inflammation leads to exaggerated stress response and cytokine expression in a newborn (7). As these changes persist long-term, they may undermine normal postnatal development of the brain (8), increasing the chances for subsequent ASD diagnosis. It is likely, however, that the molecular networks connecting PE and ASD are not limited to relatively non-specific inflammatory signaling. Identification of the shared pathways may aid in understanding the mechanisms of PE-dependent delay in brain development or its contribution to ASD-specific defects while providing possible therapeutic targets involved in the pathogenesis of both diseases.

To dissect the association between PE and ASD at the genetic level, we employed the Pathway Studio (www.pathwaystudio.com) knowledge database to undertake large-scale literature mining effort and integrated its results with an analysis of multiple PE and ASD expression datasets. We identified a set of PE-driven molecular triggers, possibly contributing to worsening the risk of subsequent ASD through a regulatory shift toward greater vulnerability to the development of ASD. Chromosome Y-encoded gene RPS4Y1, an inhibitor of STAT3 signaling, was highlighted as a PE-driven contributor to ASD phenotypes. RPS4Y1 warrants evaluation as a possible contributor to male predominance in ASD.

## Materials and Methods

This study utilized the following workflow. First, the molecules involved in either PE or ASD were extracted in the Pathway Studio environment; common molecules and pathways connecting with both diseases were identified. All data and analysis results were organized in a database ASD_PE. The downloadable form of these two databases is available at gousinfo.com/database/Data_Genetic/ASD_PE.xlsx. Then, each PE-specific gene was tested in expression mega-analysis performed across 13 independently obtained, publicly available ASD datasets retrieved from Gene Expression Omnibus (GEO) (https://www.ncbi.nlm.nih.gov/geo/). Thereafter, functional network analysis was performed to study the pathogenic significance of identified genes for ASD-related processes.

### Disease-Gene Relation Data

Disease-gene relation data for both ASD and PE were acquired through large-scale literature data analysis assisted by the Pathway Studio environment (www.pathwaystudio.com) commonly utilized for modeling the relationships between proteins, genes, complexes, cells, tissues, and diseases (9). Extracted relation data were uploaded in ASD_PE. For each of the genes linked to any of these two diseases, supporting references were examined and collated (ASD_PE: Ref4PE; Ref4ASD), including titles of the references and the related sentences describing the disease-gene relationship. Fisher's exact test was employed to compare the significance of the overlap between ASD genes and PE genes (https://david.ncifcrf.gov/content.jsp?file=functional_annotation.html).

### Mega-Analysis of Expression Datasets

To compile the list of gene expression datasets, a publicly available GEO database was searched using keywords “autism spectrum disorder” and “ASD,” which has returned 321 entries. This search covered the entire content of GEO and had no selection bias. Further filtering was performed according to the following criteria: (1) The data type is RNA expression; (2) the study design is case vs. control; (3) the original datasets and format files are available; and (4) the sample organism is Homo sapiens. A total of 14 datasets satisfied all the criteria listed and were pipelined into the mega-analysis of expression patterns as raw data files (Table 1).

**Table 1:** A summary of the datasets utilized in expression mega-analysis.

**Study Name**	**Dataset GEOID**	**#Control**	**#Case**	**Country**
Nishimura et al. (2007)	GSE7329	15	15	USA
Hu et al. (2009)	GSE15402	29	26	USA
Hu et al. (2009)	GSE15402	29	30	USA
Hu et al. (2009)	GSE15451	17	21	USA
Alter et al. (2011)	GSE25507	64	82	USA
Kuwano et al. (2011)	GSE26415	42	21	Japan
Voineagu et al. (2011)	GSE28521	40	39	USA
Colak and Kaya, (2014)	GSE29691	13	2	Saudi Arabia
Luo et al. (2012)	GSE37772	206	233	USA
Ginsberg et al. (2012)	GSE38322	18	18	USA
Pramparo et al. (2015)	GSE42133	56	91	USA
Griesi-Oliveira et al. (2014)	GSE62632	12	6	USA
François et al. (2014)	GSE63524	5	6	France
Liu et al. (2017)	GSE65106	38	21	USA

For across-dataset mega-analysis, the expression data were normalized and log2-transformed. The mega-analysis workflow pools individual gene expression measurements while correcting for between-study variation (10). For each expression dataset, the log-fold change (LFC) in ASD samples was calculated and used as the index of effect size. Both the fixed-effect model and random-effects model (11) were tested to study the effect size of PE-related genes on ASD, and their outputs compared. For each model, the heterogeneity of study inputs was calculated. Analyses were conducted using MATLAB (R2017a) mega-analysis package. To note, we used the term “mega-analysis” instead of “meta-analysis” to address the fact that the LFC of expression for each gene was calculated from the original datasets rather than using the values extracted from existing publications, which is the major difference between the two terms.

In mega-analysis, each gene was evaluated according to the following criteria: (1) The results were calculated from at least half of the studies; (2) *p* < 0.05; and (3) effect size (LFC) > 0.59 or < −1.00. When a gene has an effect size LFC > 0.59 or < −1.00, it means that the change in the expression level of the gene had increased by more than 50% or decreased by more than 50%. While ASD_PE→Mega-analysis presents mega-analysis outputs for the entire set of the analyzed genes, here we will discuss only the genes that satisfy the significance criteria outlined above.

### Gene Set Enrichment Analysis (GSEA) and Shortest Path Analysis

To gain functional insights into genes implicated both in ASD and in PE, we conducted a gene set enrichment analysis (GSEA) in the Pathway Studio environment, with enrichment *p*-values being corrected according to the Benjamini–Hochberg procedure (12). In addition to GSEA, for a gene showing significance in the mega-analysis, which was not yet described as an ASD contributor, a literature-based functional pathway analysis was conducted using the “Shortest Path” module of Pathway Studio (www.pathwaystudio.com).

### Multiple Linear Regression Analysis

A multiple linear regression analysis was employed to study the possible influence of the following three factors on the gene expression change: sample size, population region, and study date. *P*-values and 95% confidence interval (CI) were reported for each of these factors. The analysis was performed in Matlab (R 2017a) with the “regress” statistical analysis package.

## Results

### Common Genes for PE and ASD

As could be seen in the ASD_PE database, a total of 1,188 genes were associated with PE (see ASD_PE: PE_Genes and Ref4PE), and a total of 624 genes were associated with ASD (ASD_PE: ASD_Genes and Ref4ASD), with a significant overlap (*N* = 156, Fisher's exact test *p* = 3.14E−67; ASD_PE: common genes). To investigate the pathways shared by PE and ASD, a set of 156 common genes associated with both ASD and PE was submitted to a Gene Set Enrichment Analysis (GSEA) executed by using Pathway Studio. A total of 116 out of these 156 genes were shared among the top 10 most significantly enriched pathways (*p* < 1.20E−31, *q* = 0.05 for FDR), which are presented in Table 2. The full 94 pathways/gene sets enriched with *p* < 1.00e−20, which encompassed 144 out of 156 genes, were presented in ASD_PE→Common Pathways. Notably, a majority of the shared pathways highlighted by the GSEA approach were related to responses to some kind of external stimuli, including toxic ones, and to various aspects of the migration of cells. For detailed information regarding these significantly enriched pathways, please refer to ASD_PE→Common Pathways.

**Table 2:** Genetic pathways enriched with 156 genes contributing to both preeclampsia and ASD.

**Name**	**GO ID**	**# of Entities**	**Overlap**	***p*-value**
GO: response to toxic substance	0009636	634	52	4.86E–38
GO: response to extracellular stimulus	0009991	761	53	1.61E–35
GO: response to nutrient levels	0031667	730	52	2.16E–35
GO: positive regulation of locomotion	0040017	666	49	8.86E–34
GO: positive regulation of cell motility	2000147	630	48	8.87E–34
GO: aging	0016280	493	44	1.43E–33
GO: positive regulation of cell migration	0030335	603	47	1.47E–33
GO: positive regulation of cellular component movement	0051272	650	48	2.4E–33
GO: regulation of response to external stimulus	0032101	977	55	6.88E–33
GO: regulation of secretion	0051046	985	54	1.25E–31

*For each pathway/GO term, the p-value was calculated using the Fisher exact test against the hypothesis that a randomly selected gene group of the same size (N = 156) can generate a similar or higher overlap with the corresponding pathway/GO term. All these pathways/GO terms passed the FDR correction (q = 0.05)*.

**Figure 1:**
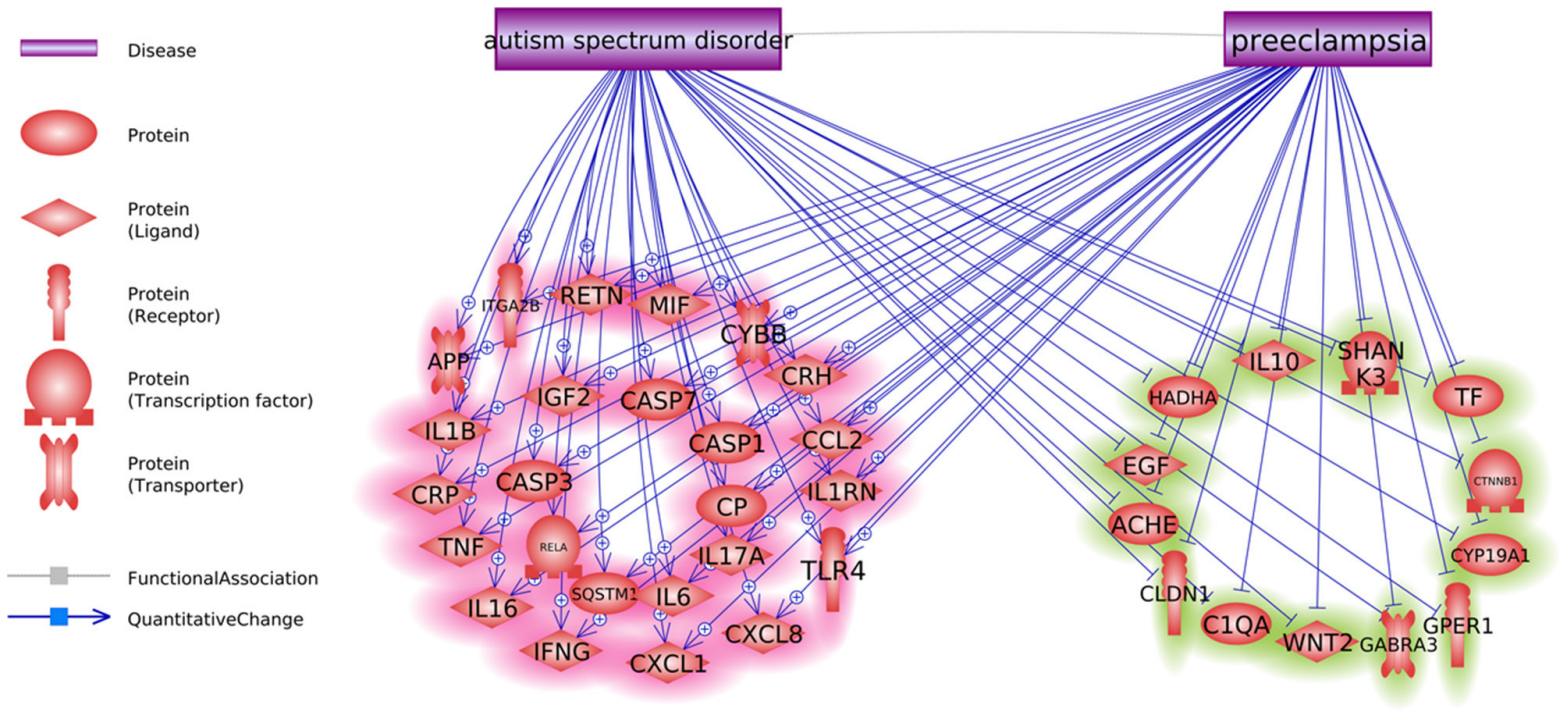
Co-directional regulatory influences exerted by PE and ASD.

We also identified six positive regulators for ASD that have been stimulated by PE condition and one inhibitor (IGF1) that has been deactivated during PE (Figure 1). The detailed information of the pathway presented in Figure 1 can be found in ASD_PE→Prognostic pathway, including the type of the relationship, supporting references, and related sentences from the references where the relationship has been identified.

### Co-directionality of the PE and ASD Phenotype Interaction

**Figure 2:**
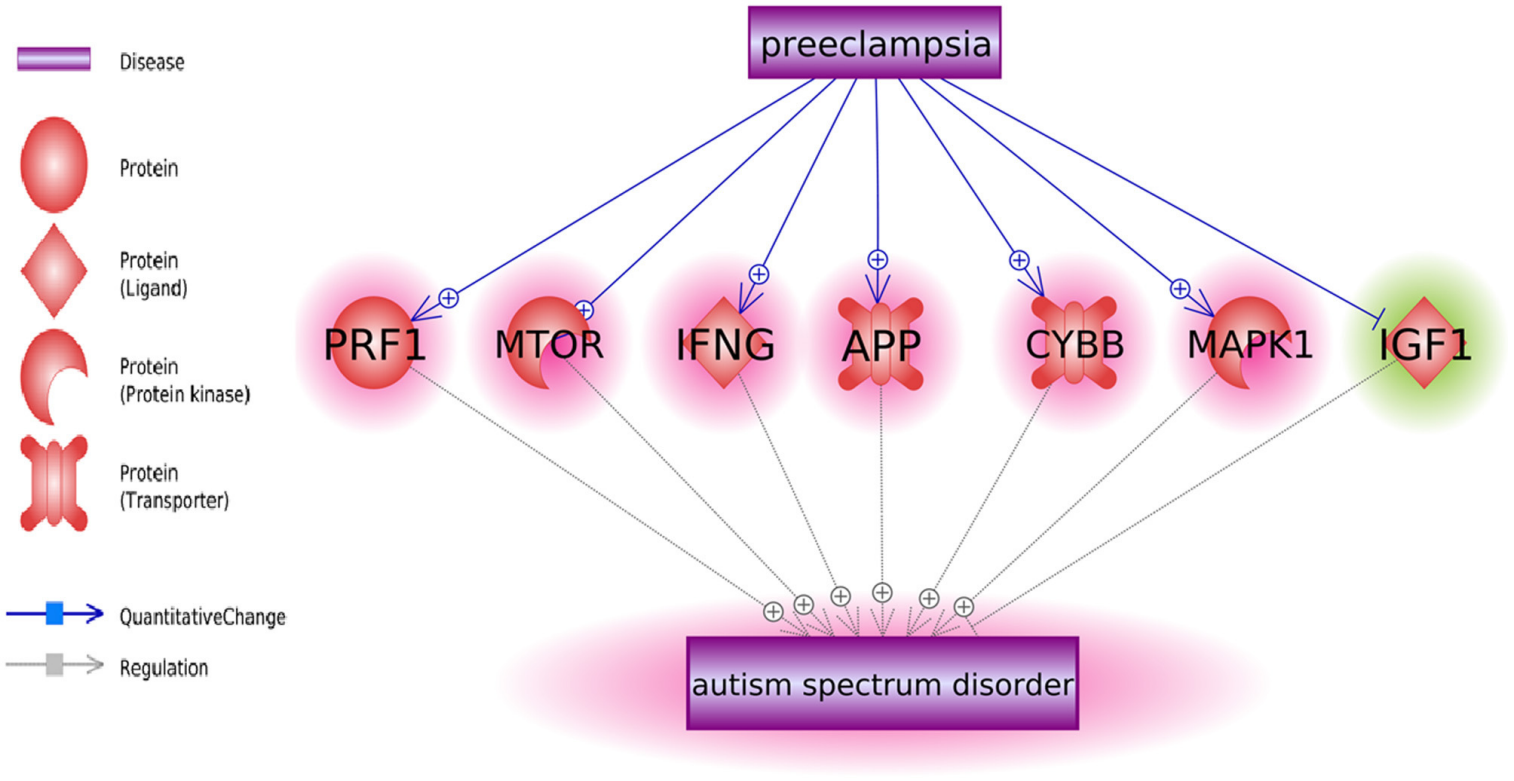
The set of molecular triggers contributing to PE-driven regulatory shift contributing to increased risk of subsequent ASD diagnosis.

Pathway analysis has also identified multiple molecules influenced by both PE and ASD (Figure 2). Notably, these interactions were predominantly co-directional. A total of 25 molecules upregulated in ASD were stimulated in PE, and a total of 13 molecules suppressed in ASD were negatively affected by PE. The detailed information regarding the network presented in Figure 2 can be found in ASD_PE→Diagnostic pathway, with each network-related entry including the type of the relationship, supporting references, and related sentences from the references where the relationship has been identified.

### Mega-Analysis of Gene Expression Pattern Identified *RPS4Y1* as a Novel Contributor to ASD

**Figure 3:**
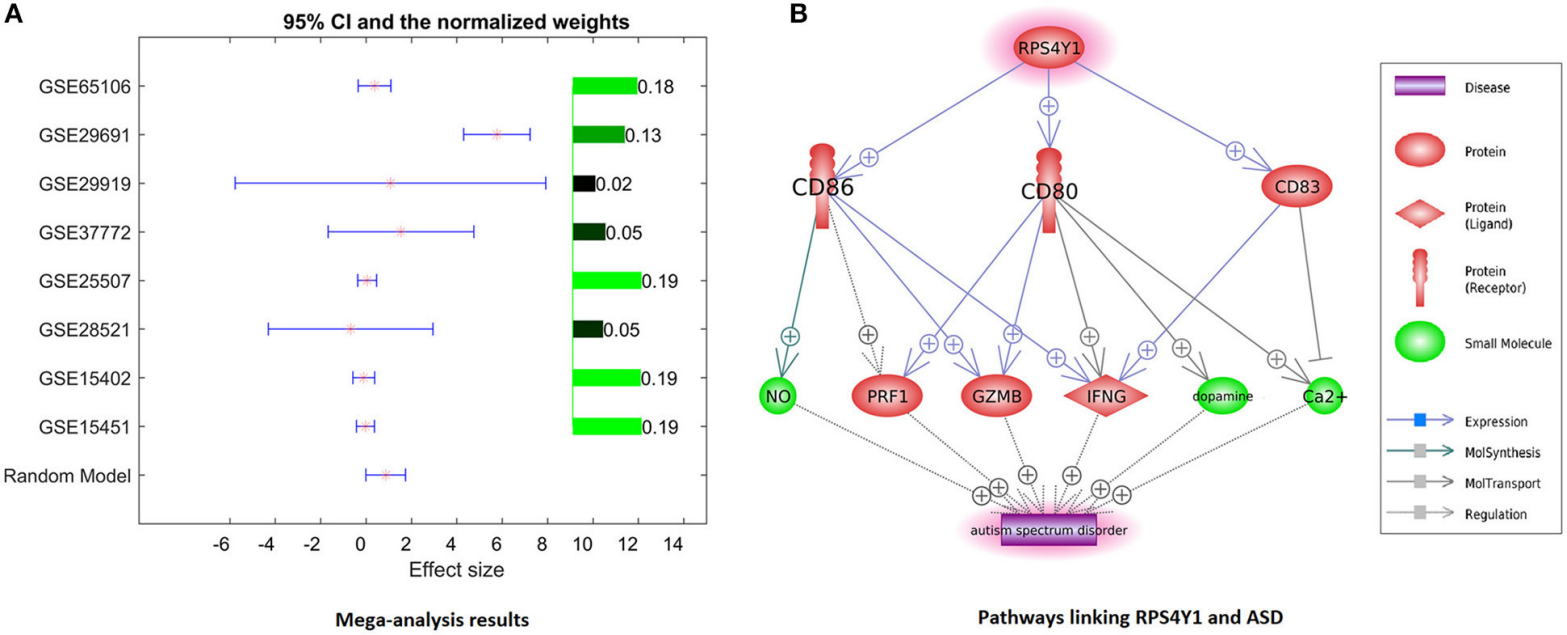
Evidence of preeclampsia-related gene *RPS4Y1* involvement in ASD phenotypes. **(A)** Dataset-specific effect sizes, 95% confidence intervals, and weights of RPS4Y1. **(B)** Molecular network connecting *RPS4Y1* and ASD.

Only one gene, RPS4Y1, has satisfied the significance criteria outlined in the Methods section as it was significantly overexpressed in ASD samples assessed in 8 out of 14 studies. To note, the other six studies have not included RPS4Y1 in the respective lists of assessed signals. Analysis of study heterogeneity showed a significant between-datasets variance of expression levels for this gene (ISq = 89.46, *p* Q > 7.74E−12), and therefore, a random-effects model was selected. Multiple linear regression analysis indicated that the expression levels of RPS4Y1 were significantly influenced by the sample size and population region (country) (*p* = 9.76E−3 and 6.19E−6, respectively), but not by the year when the study was performed (*p* = 0.94). Dataset-specific effect sizes, 95% confidence intervals, and weights of the gene are shown in Figure 3A.

## Discussion

Previous studies showed that postnatal exposure to PE is a significant risk factor for ASD with mechanistic connections being not yet described (5, 6, 13). In an attempt to identify novel, not-yet-described molecular pathways that link preeclampsia to subsequently diagnosed ASD phenotypes, we employed natural language programming environment Pathway Studio to perform large-scale exploration of previous knowledge concerning both of these conditions.

Notably, sets of genes linked to PE and to ASD demonstrated significant overlaps (see Figure 1, *p* = 3.14E−67; shared genes: *N* = 156). This set of shared genes was significantly enriched in the pathways previously implicated with both PE and ASD, such as response to nutrient levels [(14); Sharp et al., 2018], positive regulation of locomotion (15, 16), and aging [(17); Ruggieri et al., 2019]. Overall, the results of GSEA suggest that pathophysiological mechanisms of PE and ASD are partially shared.

Some molecules contributing to both PE and ASD were highlighted as molecular triggers set by PE and possibly contributing to a worsening risk of subsequent ASD (Figure 1). This set includes six ASD-positive regulators that have been stimulated by PE condition and one inhibitor (IGF1) that has been deactivated during PE. Moreover, a bulk of regulatory interactions exerted by PE condition was co-directional with that observed in ASD phenotypes. For example, in PE patients, serum levels of transferrin do decrease (18), a phenomenon suggested as a negative contributor to intrauterine growth (19). At the same time, lower than normal levels of transferrin (Chauhan et al., 2004) or the defects in its saturation (20) are commonly seen in children with ASD diagnosis (21). Similarly, levels of IL6, TNF-alpha, and IL17 are repeatedly reported as elevated in both PE and ASD [Kara et al., 2019; (22–27)].

As PE etiologically precedes the diagnosis of ASD, this finding may be interpreted as a PE-driven regulatory shift toward greater vulnerability to the development of ASD, which may occur even by quantifiable at the time of the late pregnancy or delivery (Figures 1, 2).

For further analysis, genes shared between curated PE- and ASD-specific gene sets were removed from consideration in order to ensure that uncovered PE-related contributors to ASD had not been already described as such. A total of 1,032 genes involved in preeclampsia but not in ASD were highlighted by Pathway Studio data mining. In subsequent mega-analysis of expression, each of these genes was investigated for consistent evidence of the changes in their expression in ASD phenotypes across 14 mRNA expression datasets acquired from GEO (Table 1). This analysis highlighted only one novel gene, *RPS4Y1*, which passed the preselected criterion of the significance of the association. *RPS4Y1* is a member of the S4E family of ribosomal proteins, which promotes PE by impairing STAT3 (Signal Transducer and Activator of Transcription 3) signaling and suppressing migration and invasion of trophoblast cells (28). Figure 3B shows that the product of *RPS4Y1* may also promote the development of ASD through the activation of multiple membrane proteins, including CD86, CD80, and CD83. In turn, these membrane molecules activate other molecular players previously implicated in ASD, such as ion transport pumps (29), and affect the release of nitric oxide (30). Importantly, all three of these membrane proteins promote the activity of interferon-gamma, which has been suggested to associate with increased oxidative stress in ASD (31), and also being produced at substantially elevated steady-state levels by natural killer (NK) cells of high-functioning adult individuals with ASD (32). Additional information regarding the activation of the IFNG pathway is provided in ASD_PE→RPS4Y1_Pathway.

Given the 4:1 male predominance for ASD (33), the involvement of sex-linked genes in these phenotypes is very likely. The *RPS4Y* gene is located on chromosome Y and encodes the ubiquitously expressed ribosomal protein gene. Interestingly, the expression of this gene in the process of neural differentiation increases even more (34). XY males express two RPS4-encoding genes (*RPS4Y* and *RPS4X*), whereas XX females express only one copy of *RPS4X* due to X-inactivation; thus, indicating that the levels of RPS4 protein in males vs. females are greater to begin with (35). In XYY individuals, known to score significantly higher on various autism-related scales, *RPS4Y* is expressed at the levels approximately 2-fold of that in age-matched, typically developing male controls (36). Exposure to PE may be viewed as yet another way to increase the expression of RPS4Y in affected fetuses systemically. Interestingly, Y-chromosome harbors another autism-related gene, *NLGN4Y*, which encodes a trans-synaptic cell adhesion molecule that stabilizes excitatory and inhibitory synaptic activity. Gene dosage of *NLGN4Y* is defined in a manner similar to that of *RPS4Y*. The involvement of *NLGN4Y* in autistic phenotypes is widely discussed, as it has been found mutated in some rare family clusters of autism (37). It is likely that the genetic contribution to the predominance of autistic males depends on more than one gene, thus justifying the addition of *RPS4Y*, which is normally expressed at the very high levels throughout human tissues, including the brain, to the list of the candidates in need of further exploration.

There were several limitations of this study that need to be addressed in future work. First, the expression datasets employed in the analysis were lack of age and sex information. The influence of age and sex on the identified ASD potential genes should be explored with relevant data. Second, the pathways connecting RPS4 and ASD were built based on literature data. Experiment data were needed to confirm these relations in the case of ASD (e.g., using co-expression analysis to confirm the gene–gene relations within the pathway).

## Conclusion

In conclusion, a large-scale literature mining effort and analysis of expression data allowed us to identify a set of PE-driven molecular triggers, shifting the developing brain toward a risk of ASD. In particular, chromosome Y-encoded gene RPS4Y1, an inhibitor of STAT3 signaling, was highlighted as PE-driven contributor to ASD phenotypes. RPS4Y1 warrants evaluation as a possible contributor to male predominance in ASD.

## Data Availability Statement

Publicly available datasets were analyzed in this study. This data can be found here: gousinfo.com/database/Data_Genetic/ASD_PE.xlsx; www.ncbi.nlm.nih.gov/geo/.

## Author Contributions

QX, HC, and FZ designed the study and collected the data. AB, SZ, QX, ZL, HC, and FZ performed the data analysis and contributed to the writing of the manuscript. AB and SZ provided functional analysis of RPS4Y1 and edited the manuscript into its final shape. QX and FZ contributed to the acquisition of funding that supported this study. All authors read and approved the final manuscript.

## Conflict of Interest

The authors declare that the research was conducted in the absence of any commercial or financial relationships that could be construed as a potential conflict of interest.
